# Factors Affecting Parent-Adolescent Discussion on Reproductive Health Issues in Harar, Eastern Ethiopia: A Cross-Sectional Study

**DOI:** 10.1155/2014/102579

**Published:** 2014-05-29

**Authors:** Tesfaye Assebe Yadeta, Haji Kedir Bedane, Abera Kenay Tura

**Affiliations:** ^1^School of Nursing and Midwifery, College of Health and Medical Sciences, Haramaya University, P.O.B. 235 Harar, Ethiopia; ^2^Department of Public Health, College of Health and Medical Sciences, Haramaya University, Harar, Ethiopia

## Abstract

*Background.* Open family discussion on reproductive health (RH) issues often leads to increased awareness on RH matters and reduces risky behaviors among adolescents. This study was conducted to assess factors affecting parent-adolescent discussion on RH issues in Harar, Ethiopia. *Methods.* A cross-sectional survey using face to face interview supplemented with focus group discussion (FGD) was conducted on 751 randomly selected parents of 10–19-year-old adolescents. Data was analyzed using SPSS version 15. *Results.* More than one-fourth (28.76%) of parents reported discussing RH issues with their adolescents during the last six months. In the logistic regression, parents who have demonstrated good RH knowledge and positive attitude towards RH were almost six times and seventy percent (AOR 5.69, 95% CI: 3.67–8.82; AOR 1.70, 95% CI: 1.08–2.68) higher in discussing RH with their adolescents than their counterparts, respectively. *Conclusion.* Parent-adolescent discussion about RH issues rarely occurs and is bounded by lack of knowledge, sociocultural norms, and parental concern that discussion would encourage premarital sex. Reproductive health programs should target on improving awareness of parents and addressing sociocultural norms surrounding reproductive health issues.

## 1. Introduction 

Many adolescents often lack strong and stable relationships with their parents or other adults which are necessary to openly discuss reproductive health concerns. Therefore, many teenagers do not have access to reliable information regarding their RH needs. In most cultures, parents and family members are an influential source of knowledge, beliefs, attitudes, and values for children and young people. Parents often have the power to guide children's development in sexual health matters, encouraging them to practice reasonable sexual behavior and develop good personal decision making skills [1–3]. Researches indicated that increased parent-child communication leads to a raised awareness and reduction in risk taking behaviors [4]. However, when young people feel unconnected to home and family, they may become involved in activities that put their health and wellbeing at risk [1, 5, 6].

Even though parents are main sources of information on RH issues, there is silence between most parents and their adolescent children on these matters. Studies have shown that only 46%, 20%, and 20% of parents in USA, Lesotho, and Ethiopia, respectively, had discussed such issues with their adolescents. In China, only one-third of female youths talked to their mothers about sexual matters [1, 7–9]. As a result, most adolescents' patchy knowledge on RH issues often comes from information shared by their same sex peers, who may or may not be well informed. This can lead to misinformation and the persistence of damaging myths, making young people vulnerable to unprotected sex, unwanted pregnancy, sexually transmitted diseases, and unsafe abortions [9].

The consequences of adolescent sexual behavior are an enormous burden both for the adolescent and for society. The problem is not that teens are sexually active but rather that they are inadequately prepared and guided in developing responsible sexual behaviors. The family, the major socializer of other behaviors, is not powerful in shaping adolescents' sexual behavior because of the socio-cultural, religious beliefs and sense of morality barriers affecting discussion of sexual and reproductive health issues with their adolescents [7–9]. Factors affecting parent-adolescent discussion of a country should be taken into account during planning of RH programs. Therefore, this study was conducted to assess factors that affect parent-adolescent discussion on RH issues with their adolescents in Harar town, Harari region, Ethiopia, in May 2010.

## 2. Methods

### 2.1. Study Area and Period

The study was conducted in Harar town, capital city of the Harari National Regional State, located 525 KM away east of Addis Ababa. According to the 2007 census the projected population of the town in 2010 is estimated to be 107,531. The town is divided into six woredas (districts) with a total of 19 kebeles (the smallest administrative unit). In the town there are two government, one military, one police, and two private hospitals and eight health centers. There is also one youth clinic which provides adolescent health services under the Family Guidance Association of Ethiopia. The study was conducted from April to May 2010.

### 2.2. Study Design

The study was a cross-sectional design in nature using a mixed method in the view of the sensitivity and complexity of reporting sexual and reproductive health [10]. A qualitative method was used to better understand about underlying behaviors, attitudes, perceptions and culture about reproductive health from perspectives of parents and adolescents [11, 12]. The quantitative study was conducted before the qualitative one. Findings from the qualitative study were used to triangulate that of the quantitative data. The participants for the qualitative studies were selected purposively and were not participating in the quantitative study.

### 2.3. Population, Sample Size, and Sampling Technique

The source populations for this study were all parents living in Harar having a child between 10 and 19 years of age (adolescent). All parents/guardians living in the selected kebeles of the randomly selected woredas were the study population. The sample size was estimated based on the assumption of a 20% discussion rate in Ethiopia [7], a 4% margin of error, a 95% confidence level, and design effect of 2. Multistage sampling technique was used for this study. The population was stratified by woredas (district). The town has six woredas. From each woreda one kebele was selected by simple random sampling technique. The number of households to be included in each kebele was determined in proportion to the total number of households in each kebele. A systematic sampling method was then employed to select the households and household heads (parents) were interviewed based on the objective of the study. In case no eligible candidate was identified in a selected household, the interview was conducted in the next household where there was eligible candidate. The participants of the qualitative study were selected purposively. Selection of parents was made as per the recommendation of school directors and health extension workers working in the community based on their involvement of school affairs in the school-community relationship and community health activity. The adolescents were selected from the Youth Center of the Family Guidance Association of Ethiopia's Model Clinic.

### 2.4. Data Collection

Trained nurses have collected the data by face to face interview using a structured standard questionnaire that was previously used in Ethiopia using the local language (Amharic). The questionnaire consisted of sociodemographic characteristics, knowledge about RH, attitude towards reproductive health, and discussion of RH related question. The interview was conducted in a private place and under supervision of the principal investigators. Sociodemographic characteristics relating to parents knowledge, attitude, sociocultural norms, time constraints, and perceptions of initiating discussion about sex were used as independent variables, whereas discussion was used as dependent variable. The data collectors were trained for two days in the objective of the study, the handling of study participants, and other ethical issues. A discussion guide adapted from previous similar studies was used to lead the qualitative data collection with probing as appropriate. It was collected by the principal investigators. The FGD for females was moderated by a female moderator with master's degree and similar experience to ease discussion among the females.

### 2.5. Data Processing and Analysis

After cleaning and checking the questionnaire for completeness, data was entered into computer and SPSS version 15.0 was used to analyze the data. The results were summarized by using descriptive statistics, together with an odds ratio with 95% confidence intervals and multiple logistic regressions were employed to describe the strength of association between the selected study variables by controlling the effect of possible confounders. Knowledge of reproductive health issues was assessed using a set of 20 standard questions and average score was calculated after the responses have been coded. Respondents who scored more than average were considered as having good knowledge while the remaining participants were classified as having poor. Analysis of the qualitative data was accomplished based on the predetermined themes and adding the context of additional information provided by the respondents. The data was then organized and each issue was discussed independently.

### 2.6. Ethical Consideration

Ethical clearance for the proposed research was obtained from Haramaya University College of Health and Medical Science Institutional Research Ethics Review Committee (IRERC) and informed consent from every participant was obtained before the interview. Participants were informed about the objective of the study and their rights. Both the interviews and FGD were conducted in separate private places. Codes were used for identifying FGD participants.

## 3. Results

### 3.1. Sociodemographic Characteristics of Parents

From total of 768 parents identified, 751 (97.8%) parents had participated in the study and 17 (2.2%) parents were nonrespondents due to different reasons. The nonrespondents have no sociodemographic difference from the respondents. The mean age of respondents was 44.8 years (SD 11.1). Majority of the respondents were females (76.3%), housewives (50.2%), and Orthodox Christians (56.5%) and are illiterate (21.84%) (Table 1).

### 3.2. Knowledge and Attitude towards Reproductive Health

**Figure 1:**
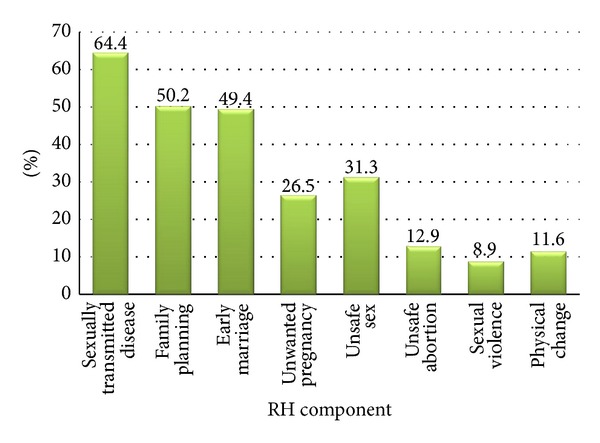
Parents' knowledge of adolescent reproductive health components in Harar town, Harari region, Ethiopia, May 2010.

Respondents' knowledge of RH issues was assessed by asking a set of closed ended questions adapted from previous study. Respondents were asked if they knew RH and 506 (67.38%) of them reported they knew what it means. Specific components of RH mentioned by the parents included STD (64.45%), family planning (50.20%), and early marriage (49.40%) (Figure 1). When asked about consequences of unprotected sex, the majority (90.01%) mentioned STD followed by unwanted pregnancy (64.85%) and unsafe abortion (38.75%). Only 14% of the respondents correctly mentioned the correct age for early marriage in Ethiopia. Responses about behavioral and physical changes during puberty showed, 701 (93.34%) breast enlargement and 703 (93.61%) beginning of menstruation in females and change in voice for males (89.35%). After all the responses to knowledge have been summed up and scored, 268 (35.68%) of the respondents scored below average and demonstrated poor knowledge of RH issues.

Attitude of parents towards discussion on RH issues was measured by a set of questions using the Likert scale. The majority of the parents (94.14%) agreed on the need to discuss RH matters. Parents should encourage their adolescents to ask questions related to their RH needs as reported by 88.75% of the respondents. However, around 21.84% of parents think that discussion with adolescents will make them promiscuous and only 16.11% of parents approved the use of family planning by their adolescents. In general a combined score for the four questions indicated that 604 (80.43%) of parents had positive attitude towards reproductive health and its discussion.

### 3.3. Discussion on Reproductive Health Issues

**Figure 2:**
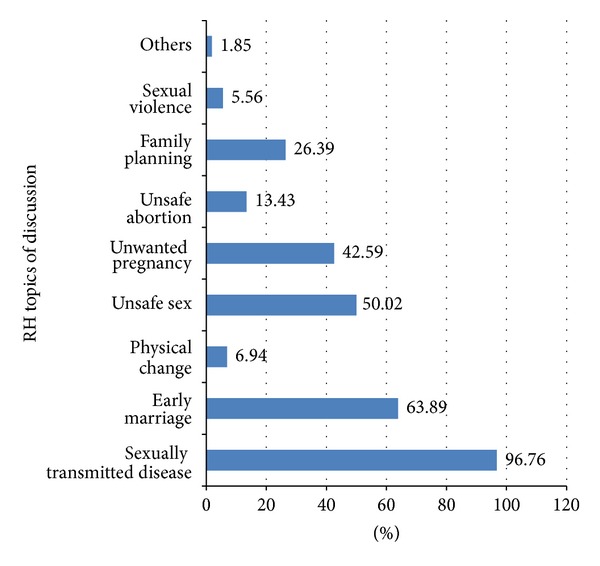
Distribution of common reproductive health topics discussed between parents and their adolescents in Harar town, Harari region, Ethiopia, May 2010.

**Figure 3:**
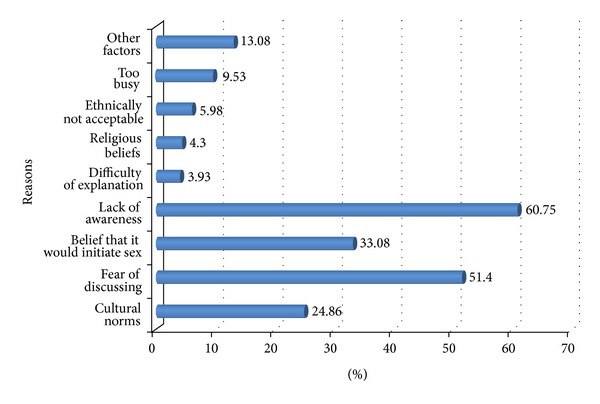
Reasons for not discussing RH matters between parents and adolescents in Harar, Ethiopia, May 2010.

Overall, only 216 (28.76%) of the respondents had reported discussion about at least two components of RH matters in the last six months prior to the study. It was found that most of the parents who reported discussions with their adolescents were males (35.92% versus 26.52%) compared to females. The study indicated that the majority of female respondents prefer to discuss with their daughters (50.7%) while male parents preferred to discuss with both boys and daughters (53.1%). The major topics of the discussions were STD, 209 (96.76%); early marriage, 138 (63.89%); unsafe sex, 108 (50.02%); and unwanted pregnancy, 92 (42.6%) (Figure 2). The most common reason for not talking with adolescents is lack of awareness of RH (60.75%) followed by the fear of discussion (51.40%) and perceiving that it would initiate adolescent's sexual practice (33.08%) (Figure 3).

### 3.4. Summary of Focus Groups Discussion with Parents and Adolescents

A total of 35 discussants participated in the four FGDs conducted with both parents and adolescents. Males and females from both groups have participated in different groups. In the two FGDs conducted with parents, similar to the findings from the survey, the majority of the parents have shown positive attitude towards importance of discussing RH matters with their adolescents. In the discussion most of the parents think that their adolescents are not sexually active and in practice only few of them have reported discussing the matter. Most of the parents participated in the discussion think that they have limited knowledge about RH so that they are unable to initiate discussion regarding the RH matters. This is evident from the response, “*We are supposed to tell our adolescents everything that has to do so with reproductive health. But I do not feel that we know all information they need,*” a 50-year-old male discussant. Parents are also found to be more directive and strict about the information to tell or not. This is evidenced by the response of one discussant,* “I have to tell my daughter only the information that I think are important to her because she will become more promiscuous if she received more information about it,*” a 39-year-old female parent. Feeling ashamed by parents and/or their adolescents is another reason stated as challenging the occurrence of discussion between parents and adolescents:* “I cannot discuss with my child about sex because it is shame for me. He will not understand me as he feels shame too,” *a 42-year-old male parent. From the discussants, only one parent has clearly refused to do so:* “If I start to discuss with my adolescent children about this issue, it means that I am saying that this is the way to…,”* a 46-year-old female parent. The issue of discussing RH issues is bound by several sociocultural norms and expectations: “*In our culture discussing about sexual matter is rare. Let alone discussing with your child, husband-wife discussion on this issue is not practiced. Everybody is shy about it. These culture, taboo and traditions are passing from generation to generation. We were brought up like this and are doing it today,”* a 56-year-old male parent.

Similar findings from the FGD conducted with adolescents indicated that discussion of reproductive health issues rarely occurs between them and their parents. Adolescents reported different factors affecting this event. The reason they mentioned were as follows: parents are not knowledgeable; they prefer to discuss with peers and at school with their teacher; they consider it as sociocultural taboo; they worried about their parents' reaction; and the parents will think they have had sex or are going to have sex. They consider their parents as not knowledgeable about the subject matter: “*Both my mother and father are illiterate, so for what topics I am talking about?” *A 17-year-old adolescent replied and said that he prefers to discuss with his peers. Some of the respondents also strengthened the effect of cultural norms preventing them from talking with parents: “*There is no trends and experience for discussion on such issues in my family and even in the community since it is considered culturally unacceptable and socially embarrassing,*” a response of a 15-year-old male youth. The fear about reaction of their parents towards their request or need for discussing the issue is also mentioned by most of the female discussants: “*If I will ask my parents about what is this and what to do about, they will consider my request as if I would be engaged in the activity,” *a 14- year-old son.* “When I asked my mother about what could cause changes as breast enlargement, she told me that it is nothing and told me to focus on my studies,” *an 18*-*year-old* female discussant, *indicating that adolescents are not given right responses even on request. Most of the adolescents also indicated that parents prefer to discuss RH indirectly by taking impersonal examples than referring to their child. This is evident by the response given by 19-year-old girl: “*You know Ms X's daughter. She has got HIV from going to many boys, she is a bad girl. Please keep your family's reputation*”. Adolescents also indicated that parents want to be more directive, monitoring rather than creating an open environment in which adolescents are asking and getting responses as required.

### 3.5. Factors Affecting Parent-Adolescent Discussion on Reproductive Health Issues

As shown in Table 2, the probability of discussion was found to be significantly higher among parents who had completed some form of education compared with parents who had no formal education: grades 5–8 (AOR 3.79, 95% CI: 1.87–7.71), grades 9-10 9(AOR 8.74, 95% CI: 3.91–19.56), and grade 10 and above (AOR 17.35, 95% CI: 8.2–36.63). Parents who reported a monthly income of above 1000 Ethiopian Birr had a twofold increase in the odds of discussing RH issues with their children (AOR 2.14, 95% CI: 1.12–4.00) compared to parents with a monthly income less than 400 Birr. Housewives demonstrated a 50% lower tendency to discuss RH issues compared with government employees (AOR 0.48, 95% CI: 0.28–0.84). Parents who have demonstrated good RH knowledge and positive attitude towards RH are almost six times and seventy percent (AOR 5.69, 95% CI: 3.67–8.82; AOR 1.70, 95% CI: 1.08–2.68) higher in discussing RH with their adolescents than their counterparts, respectively.

## 4. Discussion

Adolescence is a developmental period marked by sexual discovery and often by sexual risks. A principal means for transmitting sexual values, beliefs, expectation, and knowledge between parents and their adolescents is through discussion. This discussion is most likely to promote healthy sexual development and reduce sexual risks when parents are open, skilled, and comfortable in their discussion of sex-related topics. This study has examined the occurrence of parent-adolescent discussion of reproductive health issues and factors affecting its occurrence.

Though there is evidence that teenagers prefer to receive information about sexuality from their parents, in reality few have this privilege [13]. Similarly, in this study, even though the majority of the parents approved the importance of discussion, only 28.76% of parents have reported discussing RH issues. This is also true in other studies conducted in Ethiopia and abroad [6, 7, 9, 14, 15] which reveal that the discussion rarely occurs despite accepting its importance. But it is lower than other findings from Hawassa and Nekemte, Ethiopia [16, 17] and USA [18].

The greatest proportion of parents has participated in discussion about STDs. This finding is similar to other studies conducted in Ethiopia [14–16]. It can be argued that most parents are focusing on the negative aspects of RH rather than working on the preventive aspects. Parents who attended higher level and primary level education were more likely to discuss reproductive health issues with their adolescent children when compared to parents who received no formal education. This finding is consistent with the studies conducted in USA and selected regions of Ethiopia [2, 16–18].

Parents also indicated various reasons highlighting why they do not discuss reproductive health issues with their children. The majority (60.7%) of respondents claimed lack of awareness regarding RH issues as a reason followed by difficulty to initiate discussion due to fear and shyness (51.40%). Twenty-five percent (24.9%) of parents worried about their culture, which was a lower proportion of the total respondents than the study conducted in other administrative regions of Ethiopia [2, 16]. However, it is similar to studies conducted in China, Kenya, and Sweden [1, 6]. In addition, 33.1% of parents perceive that discussing sexual matters with their adolescents might encourage the children to engage in premarital sex. This finding is proportionately lower than the findings of the study conducted in the selected region of Ethiopia [2].

This finding from the FGD showed that parents have realized the importance of discussing RH issues with their children. But many of the parents indicated that they are unable to do so because it is culturally unacceptable; they cannot deliver the subject because of lack of knowledge or they felt embarrassed. The majority of the parents also reported that their adolescents might not take it seriously and felt that they could not answer their question regarding RH or STD/HIV/AIDS. This finding is true also in the studies conducted in Ethiopia and Nigeria [7, 15–17, 19]. Findings from the FGD with adolescents have indicated the importance of parents' involvement in socialization of adolescents about sexuality and RH. However, most adolescents prefer to discuss with their peers rather than their parents because they think that their parents are not knowledgeable about the subject matter or they may face challenges since it is embarrassing for both the adolescents and their parents. This finding indicates that adolescents are seeking advice from individuals who are knowledgeable or are not decision maker in the adolescent's life.

## 5. Conclusion 

Evidences indicated that supportive communication between parents and children enables young people to make a safe and confident transition to adulthood. But in this study the proportion of parent-adolescent discussion about reproductive health was found to be low and is bound by traditional norms, lack of information, and limited skills of discussion and creating supportive environment for adolescents. Most of the adolescents who participated in the FGDs thought their parents have no knowledge about RH issues and prefer discussing with their peers. Parents should be equipped with essential RH information for improving their discussion skills. The sociocultural norms and traditions about discussion on RH among families should be considered for better RH outcomes.

## Figures and Tables

**Table 1:** Sociodemographic characteristics of parents, Harar town, Harari region, Ethiopia, May 2010.

Variables	Frequency	Percentage
Age		
<35 years	125	16.60
35–45 years	334	44.50
>45 years	292	38.90
Sex		
Male	178	23.70
Female	573	76.30
Family size		
1-2	194	25.83
3–5	435	57.92
>5	122	16.25
Ethnicity		
Oromo	175	23.30
Amhara	350	46.60
Harari	135	17.98
Others	91	12.12
Religion		
Orthodox	425	56.59
Muslim	255	33.95
Protestant	68	9.05
Others	3	0.41
Marital status		
Married	571	76.03
Single	4	0.53
Separated	42	5.59
Divorce	34	4.53
Widowed	100	13.32
Educational level		
Illiterate	164	21.84
Read and write only	61	8.12
Grades 1–4	60	7.99
Grades 5–8	182	24.23
Grades 9-10	70	9.32
Above 10	214	28.50
Occupation		
Government employee	184	24.50
Housewife	377	50.20
Unemployed	27	3.60
Merchant	93	12.38
Others	70	9.32
Income		
<400 Birr	214	28.50
400–999 Birr	221	29.42
1000 and above Birr	181	24.10
Unknown income	135	17.98

**Table 2:** Predictors of discussion on reproductive health issues between parents and their adolescents, Harar town, Harari region, Ethiopia, May 2010 (*N* = 751).

Variables	Discuss with children		Crude OR (95% CI )	Adjusted OR (95% CI)
	Yes	No		
Educational level				
No formal education	13	212	1.00	1.00
Grades 1–4	6	54	1.81 (0.66, 4.99)	1.72 (0.62, 4.79)
Grades 5–8	35	147	3.88 (1.99, 7.59)*	3.79 (1.87, 7.71)*
Grades 9-10	27	43	10.24 (4.89, 21.43)*	8.74 (3.91, 19.56)*
Above 10	135	79	27.87 (14.92, 52.07)*	17.35 (8.22, 36.63)*
Occupation				
Government employee	109	75	1.00	1.00
Housewife	66	311	0.15 (0.10, 0.22)*	0.48 (0.28, 0 .84)*
Others	41	149	0.19 (0.12, 0.30)*	0.53 (0.31, 0.92)*
Monthly income				
<400 Birr	28	186	1.00	1.00
400–1000 Birr	63	158	2.65 (1.62, 4.34)*	1.26 (0.71, 2.26)
>1000 Birr	101	80	8.39 (5.12, 13.74)*	2.13 (1.14, 4.00)*
Do not know	24	111	1.44 (0.79, 2.60)	1.46 (0.75, 2.85)
Knowledge about RH				
Poor	27	241	1.00	1.00
Good	189	294	5.71 (3.69, 8.85)*	5.69 (3.67, 8.82)*
Attitude towards discussing RH				
Disagree/indifferent	30	117	1.00	1.00
Agree	186	418	1.74 (1.12, 2.67)*	1.703 (1.08, 2.68)*

*P < 0.05.
